# Quality improvement in palliative care: A review of the ethics

**DOI:** 10.1177/09697330241305546

**Published:** 2024-12-20

**Authors:** Fearon David, Knights Felicity, Ratiram Cherisse, Grant Liz, Fallon Marie

**Affiliations:** 172239 The University of Edinburgh Usher Institute of Population Health Sciences and Informatics; University of Edinburgh

**Keywords:** Data protection, ethics, governance, palliative care, quality improvement

## Abstract

**Introduction:**

Quality improvement is the systematic seeking of improvements in care and experience. This discussion paper will explore how the principles of good clinical care and the established ethical frameworks for research can help guide its practice, using examples from palliative care.

**Quality improvement in palliative care:**

Palliative care is well positioned to be at the vanguard of quality improvement in healthcare. But it holds ethical particularities which require specific considerations, that are helpful for other specialities. The experiences of two improvement activities in palliative care, the Liverpool Care Pathway and Do Not Attempt Resuscitation status reviews, illustrate potential dangers of QI.

**Implications for ethical practice:**

Recommendations for ethically sound quality improvement projects in palliative care include paying attention to the burden of time, viewing informed consent as a tool, monitoring for vulnerability and coercion and transparency in the use of data. The ethics and practices in clinical encounters provide a framework for approaching consent and protecting those with palliative care needs who are deemed as vulnerable. It is explicit in palliative care that time and energy are precious and finite resources. These must be valued and respected in any quality improvement projects. Respect for beneficence and autonomy is essential to avoid coercion and for any project to be ethically sound.

**Conclusion:**

Quality improvement processes are an integral part of good healthcare practices. High ethical standards, a supportive culture, transparency and candour are needed for the promotion and sustainability of quality improvement in palliative care.

## Contribution statements

### What is known

Quality improvement is an integral part of all healthcare.

All healthcare should meet ethical standards.

### What this paper adds

Quality improvement activities risk the lack official oversight with most ethics review boards deeming it as not within their remit.

Palliative care is an ideal space to promote and demonstrate ethically sound quality improvement projects, but patients with palliative care needs require special consideration around quality improvement.

## Introduction

Palliative care has expanded globally since its modern day inception in the 1960s.^1,2^ Early practitioners and advocates wished to support those living with life-limiting illnesses to cope better and die without or in less pain. They were also motivated by the prevalent attitudes and common practices which they perceived as unethical, such as the non-disclosure of diagnoses or the non-treatment of symptoms. These challenges continue in many contexts across the globe, especially where palliative care is less well established.^3–5^ In higher-income contexts with palliative care integrated within health systems, different ethical dilemmas have been prominently debated, such as the principle of double effect, terminal sedation and assisted dying.^6,7^ However, there is very little published discussion on the ethics of quality improvement (QI) in palliative care. Addressing this gap, this review paper aims to demonstrate the utility of palliative care as a platform to explore the ethics of QI, explore some potential dangers of QI and provide suggestions for undertaking ethically sound QI projects in palliative care.

### Quality improvement

Quality improvement (QI) in healthcare is the ‘systematic continuous approach that aims to solve problems in healthcare, improve service provision, and ultimately provide better outcomes for patients’.^8^ QI methodology is situated centrally between audit, quality assurance and service evaluation on one side and research on the other. While not synonymous with the evaluative methodologies, QI can be understood as an umbrella term under which these practices can be viewed as components or tools of QI, but which do not express the whole essence of it. For example, a single cycle audit does not fit within our understanding of QI; however, a two-cycle audit with the implementation of an intervention is one of the simplest examples of QI.

The relationship between QI and research is more nuanced. In many iterations of QI, knowledge established from research is simply employed to improve care. For example, QI projects introducing evidence-based clinical guidelines for specific clinical scenarios or employing broader interventions shown by research to be effective, such as the introduction of condition-specific multi-disciplinary team meetings to improve end-of-life care.^9^ In many QI projects, there is the creation of new knowledge.

There is much debate and many attempts to distinguish between QI and research.^8,10,11^ In practice, there is much uncertainty and lack of consistency in how these are applied internationally.^12^ This debate is not just theoretical. It is important because of the procedures for governance and ethical oversight. Research studies require approval from an ethical review committee, whereas QI projects traditionally do not. Despite this broad exemption, QI practitioners must consider the ethics of any project, with oversight provided as appropriate.^13^

### Quality improvement in palliative care

Palliative care is an ideal context for being at forefront of QI. It is a relatively young speciality, small enough to be flexible, interprofessional, holistic and not restricted to a single-system biomedical approach.^14^ However, applying QI methodologies within palliative care merits special ethical consideration because it is an emotive subject for staff, patients, families, communities and society. This is illustrated in the brief critique, through the lens of QI methodology, of two examples of ‘projects’: the Liverpool Care Pathway for the Dying (LCP) and Do Not Attempt Cardio-Pulmonary Resuscitation (DNACPR) orders.^15,16^

### The Liverpool Care Pathway for the dying

The LCP was a structured set of guidelines, documentation and alerts designed to improve end-of-life care in hospitals, nursing homes and occasionally at home.^15^ Prominent in the United Kingdom (UK) in the 1990s and early 2000s, it attempted to transfer a tried and tested, successful evidence-based approach from a UK hospice context to non-palliative care specialist contexts.^17^ The experience of its implementation and use in the UK has been extensively discussed in academia, government committees, healthcare organisations and the media. It is now acknowledged that multiple components of it were performed unethically, with infringements against each area of Beauchamp and Childress’ bio-medical ethical framework, justice, autonomy, beneficence and non-maleficence,^10,18^ and against many healthcare regulatory bodies’ standards of good care. For example, the lack of consent process and the inadequate communication with families illustrated a lack of respect for autonomy, and the withholding of basic care such as oral fluids was a failure to prevent non-maleficence.^15,19^ From a justice perspective, at least in the public eye, it was suspected that the LCP was a backdoor to euthanasia, prioritising cost savings over ethical care.^17^

### Do Not Attempt Cardio-Pulmonary Resuscitation orders

During the first wave of the COVID pandemic, attempts were made to reduce futile therapies to help protect the UK’s National Health Service (NHS). This included the review of the resuscitation status of care home residents.^16^ These efforts were widely perceived as the blanket, indiscriminate imposition of DNACPR orders. These were criticised in the media as devaluing vulnerable people, especially the older and those with learning disabilities.^20^ A further example of actions designed to improve care but instead going against ethical principles, in this case autonomy and justice.

The LCP and the DNACPR reviews were congruent with Backhouse and Ogunlayi’s definition of QI above. In the case of the LCP, the adoption and expansion of established knowledge and practice failed not because that the LCP was a bad tool but because, in QI terms, there was little monitoring of process of using the LCP, outcomes, unintended consequences and too few balance measures.

These two examples highlight the sensitivities and heightened emotion associated with the speciality of palliative care. It also points to the reasonableness and prudence of seeing those affected by life-limiting conditions as vulnerable and requiring a high degree of protection from harm. As the title of the UK’s government’s replacement to the LCP states ‘One chance to get it right’,^21^ there is a burden on care providers to acknowledge their responsibility to respect this limited and precious time for patients and their families. An ethics group deeming a project to be QI and therefore out with their remit does not justify ignoring important ethical principles.^13^ QI in palliative care needs to be methodologically and ethically sound, monitoring for unintended consequences. Governance and ethical oversight for the QI team can help ensure this.

## Areas of ethical tension in the context of quality improvement in palliative care

### Informed consent

People’s autonomy, and their control and understanding of the care provided to them and their loved ones were central to the challenges described above in the LCP and DNACPR controversies. Consent was lacking at times. Many debutant QI practitioners flinch at the concept of informed consent in QI. They feel confident that QI is only a force for better care and therefore informed consent is not required. This may be an accurate understanding for the simplest two cycle audit-based QI projects. For more complex QI projects, consent is a useful tool employed to ensure the ethical soundness of a project with protection for the participant and practitioner. However, there is a lack of international consensus on the role of consent in QI.^12^ New Zealand’s National Ethical Framework is helpfully explicit in the statement ‘Participants should be asked for their informed consent if a quality improvement activity imposes more than minimal risk’.^12^ However, as learned in the LCP and DNACPR experiences, risks are not only limited to the individual patient but also needs to be considered more holistically, including risks to society’s trust in their healthcare workers, collaborative teamwork and the reputation of specialities.

As many QI projects pose low or negligible risk, we propose that the clinical consultation provides a practical framework for the proportionate use of consent in QI. Implied consent from the patient or family is assumed in any empowering shared decision-making encounter where questions are asked, and a management plan is jointly agreed upon.^22^ Explicit verbal consent will be sought for clinical examination, even at a perfunctory level. For example, a clinician asking if they can listen to a patient’s chest or examine their ears while moving and preparing for such an examination. They may also ask consent prior to asking sensitive questions such as taking a sexual history or potentially distressing questions such as assessing cognitive function or psychotic symptoms. For more intimate examinations, the clinician is expected to provide a more detailed explanation of the process, and obtain and document explicit consent including whether a trained chaperone was offered and accepted.^23^ Interventions such as surgery require signed consent forms with documentation of the potential risks. We can see that as interventions become more invasive with larger risks and less clear risk-benefit ratios, more extensive handling and documentation of the consent process is required. This is the same for QI.

Some QI practitioners have avoided addressing consent because of their concern of tripping over the theoretical research-QI dividing line. Informed consent should not be feared in QI. Consent in negligible risk QI projects can be assumed by the patients’ general consent to treatment in the healthcare facility.^24^ More detailed consent should be sought when participation in a QI project poses more than minimal risks and inconvenience in comparison to usual routine care.^10,25^ Short surveys which provide no direct benefit to the participant may be sufficed by verbal consent, but written informed consent would be more appropriate for an interview. Such consent processes can aid the practitioner to ensure that their practices are sound, for example, as they consider the written information they will provide to the participating staff, patient and families.

### Burden of time

Time is a precious and finite resource for all of us, and especially evident in palliative care. Careful attention needs to be paid to any additional burden placed on the patient and family in a QI project. For example, the time for completion of a new patient-reported outcome scale (PROM) during routine consultations, or staff and patients being asked to complete surveys. The introduction of visual analogue scales to improve the measurement and documentation of pain would likely have limited impact on time; however, a comprehensive quality of life scale may have over 30 questions and take time and energy to complete, for both staff and respondent. There should be a clear advantage for the individual patient and not just a systems-level improvement. Very frequent completion of a long, in-depth scale would be inappropriate, but regular use of a shorter scale such as the Integrated Palliative Outcome Scale may be more acceptable for many patients.^26^ The key for the patient is to understand how their responses are used to improve their care.

It is not just the burden of time that a patient contributes to the QI process with their involvement. Their time is closely linked with their energy levels. A patient is likely to expend a large proportion of their limited energy reserves to complete a questionnaire in contrast to the QI practitioner who will have dispensed a negligible amount of their energy in asking the questions. Patients may require rest or sleep to recuperate, time away from engaging with the people and activities they value the most. Patients’ finite source of energy, and their physical, cognitive, and emotional needs must be respected in a QI project. This is especially evident in palliative care, where it is normal and natural for patients to progressively decline with increasing fatigue.

The burden of time is a commonly perceived barrier for healthcare professionals’ involvement in QI.^27^ They often feel that they lack the time to be involved in QI. It is important that any QI project incorporates a range of balance measures to capture and value these views and experiences. Balance measures are used to identify and measure potentially unintended effects of a QI project, usually checking for adverse effects. For example, monitoring the duration of consultations can detect if a project has brought about an unintended change. Another common example of balance measures are surveys and questionnaires. While these are commonly found in research, they are also important tools in QI. To avoid being classified as research, these must be focused on and be limited to the remit of the QI project. To avoid being an additional burden, they should be succinct and collect only justifiable and important data. Interviews and focus groups can also be employed in QI; however, these are more fluid and less controllable than a structured survey and so harder to remain within the boundaries of QI and avoid being research. The closer the QI methods are to those used in research and the further they move away from clinical benefit for the individual, then the more ethical scrutiny is required, with more consideration given to consent.

### Vulnerability and coercion

It is appropriate to consider that many people with palliative care needs are potentially vulnerable. The LCP and DNACPR controversies highlighted the dangers when healthcare workers do not sufficiently consider these vulnerabilities. Conversely and positively, they also show that society has a highly emotive response to any perceived trampling on these individuals. Effective and sensitive communication is central to ensuring quality of care and enhancing trust. Styles of communication need to be appropriately employed to ensure that effective two-way communication occurs, the same as in a clinical consultation. In addition, QI practitioners should plan for physical decline, changing care needs and temporary or permanent loss of capacity. We encourage that consent is weighed against the burden and risk of harm and the individual’s capacity. If capacity is in doubt, then so is consent.^28^ For the simpler QI projects, care for all patients is improved by moving it to be more in line with the established gold standard practices. Consent and capacity are less of an issue in this situation. For more complex scenarios in which non-routine data are being collected in situations where full capacity is lacking, formal review by an ethics group or QI governance group is advisable.

Palliative care can be an intense experience for people, families and staff. Relationships are often deep and rich. The Hawthorne effect, ‘the tendency for people to behave differently when they know they are being studied…’ (Davies and Shackleton, 1975: p55), can be pronounced in QI in palliative care due to the close, often prolonged involvement of the clinical team with the patients and families (Payne et al., 2007). Patients may be confused over whether the QI practitioner is a central member of their healthcare team, with a resulting shift of power in the relationship (Lawton, 2001). With this can come a sense of obligation to the staff member. This is well recognised in research in palliative care,^28^ but less so in QI.

### Data and duty of candour

There is a statutory and moral obligation that healthcare organisations provide the best care possible. This requires a framework of QI to both monitor and improve. It has been internationally accepted practice that clinicians with a legitimate relationship with patients have the right to access their data for QI projects posing minimal risk.^12,24^ This assumption is based on the view that QI processes are an integral part of good healthcare practices and therefore covered by patients’ implied consent to care.^24^ These assumptions are being challenged with the recent moves to patients having increased rights and power over their personal data, both within and out with healthcare settings. In the UK, The National Data Opt-Out was introduced in England in 2018 and its impact is slowly influencing QI processes.^29^ Although ‘clinical audit’ is out with the scope of the Opt-Out, there is nervousness around broader QI and data protection. For simplicity and to avoid uncertainty, most QI teams are either more formally addressing the consent processes or restricting themselves to using anonymised data. The exclusive use of fully anonymised data can potentially limit the QI team’s learning from the QI process and their duty of candour.

In the UK, healthcare organisations have an obligation, under the duty of candour, to disclose information of poor or dangerous care and apologise to the patient, or their advocate or family member.^30,31^ Duty of candour became statutory in the UK in 2014, after the arrival and departure of the LCP. The duty of candour remains if the patient has lost capacity since the event or has died. It is unambiguous on unintended and unexpected events that resulted in death or harm.^32^ In cases of ‘near misses’ where an event had the potential to cause harm but did not, guidance allows professional judgement.^30^ This is helpful as there is a risk that divulging such information to bereaved relatives may disrupt their grieving process.

Many QI projects involve retrospective data collection from clinical notes, and it is not uncommon for sub-standard or dangerous care to be incidentally identified.^13^ We encourage QI teams to incorporate a protocol for the management of any adverse event which caused harm or had the potential to, with clear designations of responsibilities within the team. It is hopeful that these system-level changes will help prevent similar negative experiences from happening again. But poor care is more commonly associated with person factors rather than a set of guidelines, both in exacerbating the problem-inducing culture and also failing to address issues when they arise.^31^ Individuals often feel powerless to bring about change, and fear being victimised if they speak out, even with the statutory duty of candour. Therefore, while the duty of candour is an important step in the right direction, it is essential that it is complemented with no-blame collaborative team working environment. The more recent work on the protection of whistle blowers should help further improvements, especially in situations of dysfunctional team dynamics.^33^

### Organisational ethics oversight

Health-related research requires ethical review and oversight from an official ethics committee or institutional review board. Traditionally, QI projects do not require such formal ethical review, to the extent that they are often excluded from review by such committees.^12,34^ There is therefore a shortage of places where QI practitioners can access proportionate and suitable formal ethical oversight. This standpoint maintains the flawed perception that QI projects do not have ethical implications nor require ethical oversight. Conversely, Steigler and Tung^11^ propose that unscrupulous researchers may reclassify smaller research studies as QI to avoid the bureaucratic and time-consuming process of formal ethical review.

According to Naughton et al.’s review of QI ethics, only New Zealand has an integrated framework for the ethical conduct of research and QI.^12^ Where we work, at the University of Edinburgh, Scotland, our online Masters of Family Medicine programme hosts a QI-specific ethics group for our students, of whom the majority are primary care professionals working in low- and middle-income settings.^35^ This provides ethical oversight for projects for which no alternative oversight would have been available. Elsewhere in the university, the Medical School Research Ethics Committee suggests that QI practitioners may wish to consider reclassifying their QI project as research if there is no quality improvement team or equivalent available at the project site in order to avoid the project undergoing no independent scrutiny.^36^ This advice is especially relevant for complex QI projects involving higher levels of consent or intense and time-consuming methods data collection, such as interviews. While this may be methodologically or practically suited for some projects, it can raise additional and potentially unnecessary burdens for the practitioners as the reviewers are experts in research and not QI.

All projects, whether research or QI, are expected to meet the same ethical standards, but we are not proposing that all need the same level of oversight, nor needing the same formality of ethical submission and approval. A capacity-building, sustainable and supportive approach would be preferable in many contexts, in which a team provides ethical oversight throughout the project’s duration. In the UK, there are now QI teams across all regional health boards who provide a supportive and constructive approach to helping their workforce engage in QI, with their own system of approvals both informal and formal sitting alongside training, coaching and mentorship.^37^ This will facilitate high-impact QI projects which are much more iterative in nature, and responsive to emerging data, experiences and learning along the way.^24^

## Conclusion

It is essential that research studies and QI projects are undertaken in accordance with the highest ethical standards, but these needs are not the same. Ethical oversight needs to be proportionate for QI. Larger, more complex projects like the LCP and more sensitive one like the DNACPRs require higher levels of review. This does make ethical oversight more challenging but, in many ways, difficult things reflect the real-world, lived experience of high quality palliative care. One important difference between research and QI is that research is optional with voluntary participation, whereas QI is usually an integral part of the evolution of ‘routine’ healthcare services.^24^ Consent for participation in much of QI is consistent with the implied consent for any medical context, such as for being asked questions, undergoing tests or examination in a consultation. It is an ethical and moral imperative that healthcare organisations evolve and improve the care provided over time.

Using the lens of good clinical practice, we have looked at several common issues which we hope will be useful for the healthcare practitioner considering QI in palliative care. It is neither practical nor useful to propose comprehensive answers to these issues, rather we hope that we have inspired and enriched the reflections of individuals and teams, who will hopefully aim for a higher level of ethical review or oversight.
